# Systemic Administration of Tripeptidyl Peptidase I in a Mouse Model of Late Infantile Neuronal Ceroid Lipofuscinosis: Effect of Glycan Modification

**DOI:** 10.1371/journal.pone.0040509

**Published:** 2012-07-06

**Authors:** Yu Meng, Istvan Sohar, Lingling Wang, David E. Sleat, Peter Lobel

**Affiliations:** 1 Center for Advanced Biotechnology and Medicine, Piscataway, New Jersey, United States of America; 2 Vivarium, University of Medicine and Dentistry of New Jersey – Robert Wood Johnson Medical School, Piscataway, New Jersey, United States of America; 3 Department of Pharmacology, University of Medicine and Dentistry of New Jersey – Robert Wood Johnson Medical School, Piscataway, New Jersey, United States of America; Baylor Research Institute, United States of America

## Abstract

Late-infantile neuronal ceroid lipofuscinosis (LINCL) is a recessive genetic disease of childhood caused by deficiencies in the lysosomal protease tripeptidyl peptidase I (TPP1). Disease is characterized by progressive and extensive neuronal death. One hurdle towards development of enzyme replacement therapy is delivery of TPP1 to the brain. In this study, we evaluated the effect of modifying N-linked glycans on recombinant human TPP1 on its pharmacokinetic properties after administration via tail vein injection to a mouse model of LINCL. Unmodified TPP1 exhibited a dose-dependent serum half-life of 12 min (0.12 mg) to 45 min (2 mg). Deglycosylation or modification using sodium metaperiodate oxidation and reduction with sodium borohydride increased the circulatory half-life but did not improve targeting to the brain compared to unmodified TPP1. Analysis of liver, brain, spleen, kidney and lung demonstrated that for all preparations, >95% of the recovered activity was in the liver. Interestingly, administration of a single 2 mg dose (80 mg/kg) of unmodified TPP1 resulted in ∼10% of wild-type activity in brain. This suggests that systemic administration of unmodified recombinant enzyme merits further exploration as a potential therapy for LINCL.

## Introduction

Classical late-infantile neuronal ceroid lipofuscinosis (LINCL) is a recessively inherited lysosomal storage disease (LSD) resulting from mutations in the gene that encodes the lysosomal protease tripeptidyl peptidase I (*TPP1*) [1], [2]. While most cell types accumulate autofluorescent storage material, symptoms are predominantly neurologic. Children typically present at 2–4 years of age with seizures followed by progressive loss of sight, motor skills and mental function. There is massive neuronal loss and death is inevitable, usually between ages seven and fifteen.

There is no effective treatment for LINCL. For LSDs in general, enzyme replacement therapy (ERT) is the most successful clinical approach [3], [4]. Here, a therapeutic protein corresponding to the deficient lysosomal activity, is infused into the bloodstream of patients and taken up by endocytosis and delivered to lysosomes of many cell types where it can correct the underlying metabolic defect. However, the blood-brain barrier (BBB) presents a major hurdle for delivery of therapeutic proteins to cells in the central nervous system (CNS).

Given that many LSDs exhibit neuronal storage and neurological manifestations, there is continued interest in developing clinically-applicable methods for delivery of a therapeutic protein to the CNS and both direct and indirect routes of administration have been considered. A number of recent studies in animal models suggest that ERT could be effective in treating neurological LSDs if the recombinant protein is delivered to the CNS via the cerebrospinal fluid by intrathecal [5]–[12] or intracerebroventricular [13]–[15] administration. However, these approaches are highly invasive and there remains considerable interest in developing strategies to deliver enzyme to the CNS from the bloodstream.

Neonatal intravenous (IV) ERT has been shown to improve neuropathology in mouse models of Mucopolysaccharidoses IIIA and VII [16], [17]. Here, delivery across the BBB occurs via mannose 6-phosphate (Man6-P) dependent uptake and transcytosis, but is limited to the first two weeks of life due to developmental down-regulation of the cation independent Man6-P receptor (CI-MPR) at the BBB [18], [19].

For adult animals, the efficiency by which therapeutic proteins enter the CNS is increased dramatically when there is a persistent steady-state presence of these proteins in the bloodstream. Treatment regimens employing repeated administration of high doses of enzyme resulted in delivery of enzyme to the brain and improvements in CNS pathology in several LSD mouse models whereas lower doses had little or no effect [20], [21]. Here, high doses may saturate the major clearance pathways (e.g., via hepatic CI-MPR and mannose receptors) and thus extend the presence of the therapeutic protein in circulation. In addition, carbohydrate modification of glycoproteins can inhibit clearance and has been reported to improve transit across the BBB [22]. Periodate-treated β-glucuronidase had a prolonged circulatory half-life following IV administration (hours versus minutes for the unmodified protein) and it is likely that this allowed for increased uptake to the CNS by either fluid-phase pinocytosis, endocytosis mediated by an as yet unrecognized receptor, or an “extracellular route” that allows small amounts of proteins to enter the CNS from the bloodstream. Regardless of the route of uptake, the chemically modified β-glucuronidase entered the CNS and reached activity levels of ∼8% of normal, resulting in a significant clearance of CNS storage in neocortical and hippocampal neurons.

**Figure 1:**
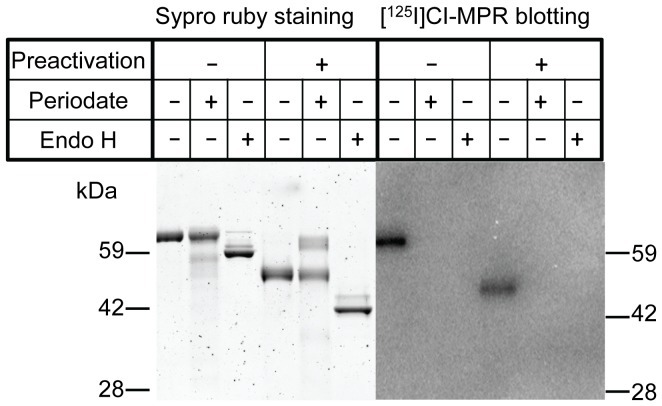
Reducing SDS-PAGE analysis of native and carbohydrate modified TPP1. Left panel: Sypro ruby stained gels. Right panel: Blots probed with soluble I^125^-CI-MPR. Note that Endo H treatment results in a shift of ∼6 KDa, consistent with removal of *N*-linked oligosaccharides.

In this study, we have investigated the influence of dosage and carbohydrate structure on the pharmacokinetics of IV administered TPP1. We find that either the removal or oxidation of glycans increases the circulatory half-life of TPP1 but these treatments do not improve uptake to the CNS. Surprisingly, we find that a significant amount of unmodified TPP1 itself does enter the brain in a dose-dependent manner.

**Figure 2:**
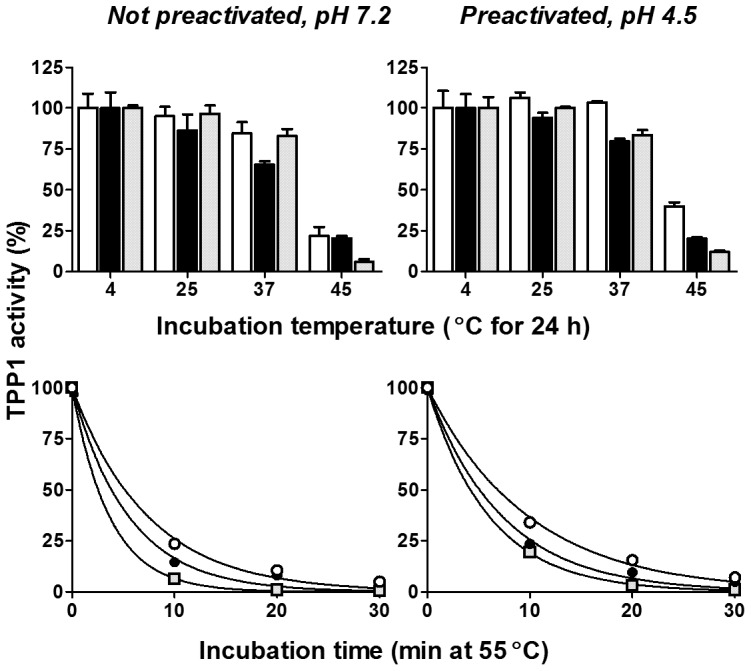
Thermostability of TPP1 preparations. *Left Panels*: Proenzyme was incubated at pH 7.2 at different temperatures and then frozen at the indicated times. Preparations were subsequently autoactivated at pH 3.5 for 1.5 h at 37°C prior to measuring enzyme activity. *Right Panels*: Proenzyme was autoactivated prior to incubation at pH 4.5 at the indicated times and temperatures and then frozen prior to directly measuring TPP1 enzyme activity. Results (mean and standard deviation of duplicate determinations) are normalized to the activity of enzyme incubated at 4°C. Unmodified TPP1, open circles and bars; OX-TPP1, filled circles and bars, DG-TPP1, gray squares and bars. The concentration of enzyme during incubation was 0.1 mg/ml.

## Results

### Carbohydrate modification of TPP1

Our overall goal was to determine whether extending the circulatory half-life of TPP1 would increase uptake into the brain, as has been observed previously for β-glucuronidase [22]. To this end, we modified TPP1 to prevent endocytosis and clearance by carbohydrate receptors (e.g., the mannose receptor and CI-MPR) in peripheral tissues using either chemical modification with periodate/borohydride to produce OX-TPP1 or enzymatic deglycosylation of high-mannose TPP1 with Endoglycosidase H (Endo H) to produce DG-TPP1. Both treatments destroyed the Man6-P recognition marker (Fig. 1) as determined using an iodinated form of the CI-MPR as a specific affinity probe [23].

**Figure 3:**
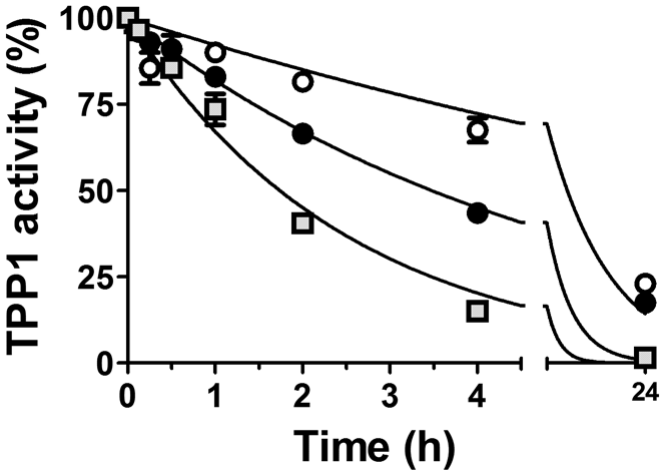
*In vitro* stability of proTPP1 preparations in plasma. ProTPP1 diluted in aCSF was mixed with plasma from *Tpp1*(−/−) mice (final concentration 0.08 mg/mL TPP1, 87.5% plasma) on ice. Time courses were started by incubation at 37°C and samples frozen at the indicated times. Preparations were then activated and TPP1 activity measured and normalized to the zero timepoint. Unmodified TPP1, open circles; OX-TPP1, filled circles, DG-TPP1, gray squares. Data was fit to a single-phase exponential decay model using Graphpad Prism constraining initial values to 100% and the plateau value to 0%, yielding the following half-life estimates (with 95% confidence limits): unmodified TPP1, 8.6 (6.7–11.9) h; OX-TPP1, 3.5 (2.9–4.3) h; DG-TPP1, 1.7 (1.5–2.1) h.

#### Enzymatic properties

TPP1 is synthesized as an inactive zymogen that is stable at neutral pH. Acidification of purified proenzyme *in vitro* triggers autocatalytic cleavage with conversion of the protein to the active, processed form similar to that found in the lysosome [24]. Both OX-TPP1 and DG-TPP1 largely retained the ability to autoactivate to the processed form (Fig. 1). The specific enzymatic activity of DG-TPP1 is essentially the same as unmodified TPP1 ([25] and data not shown). However, chemical modification resulted in a loss of ∼18% activity in OX-TPP1 (data not shown), which is likely reflected by the minor amount of OX-TPP1 that appears refractory to autocatalytic processing (Fig. 1). A small decrease in specific activity was also observed when β-glucuronidase was similarly treated with periodate [22]. Although this suggests a minor amount of denaturation or chemical modification of catalytically important residues, the OX-TPP1 retained sufficient activity to allow further analysis of its pharmacological properties.

**Figure 4:**
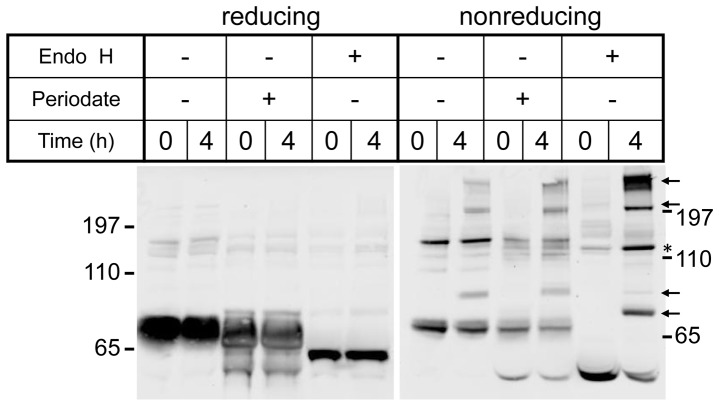
Immunoblot analysis of TPP1 preparations incubated in plasma. Incubations were conducted as in Fig. 3. Samples were heated in denaturing SDS-PAGE sample buffer that either contained or lacked 5 mM dithiothreitol as indicated (reducing or nonreducing).

#### Stability

Carbohydrate residues are believed to enhance the thermal stability of glycoproteins and their modification or removal may be destabilizing [26]. We thus investigated the effect of temperature on both the proform incubated at neutral pH and the activated enzyme at acidic pH for the different TPP1 preparations. For unmodified TPP1, both the zymogen and activated enzyme were stable at temperatures up to 37°C for 24 hours and lost significant activity at 45°C (Fig. 2). A similar trend was seen for the modified forms, although they appeared more sensitive to inactivation at higher temperatures. All forms were rapidly inactivated at 55°C, with the unmodified form being the most stable. Thus, while carbohydrate modification or deglycosylation has a demonstrable effect on TPP1 thermostability, the modified forms are moderately stable at physiological temperatures, with the OX-TPP1 proenzyme and active form retaining 65% and 80% activity, respectively, after 24 hours at 37°C.

**Figure 5:**
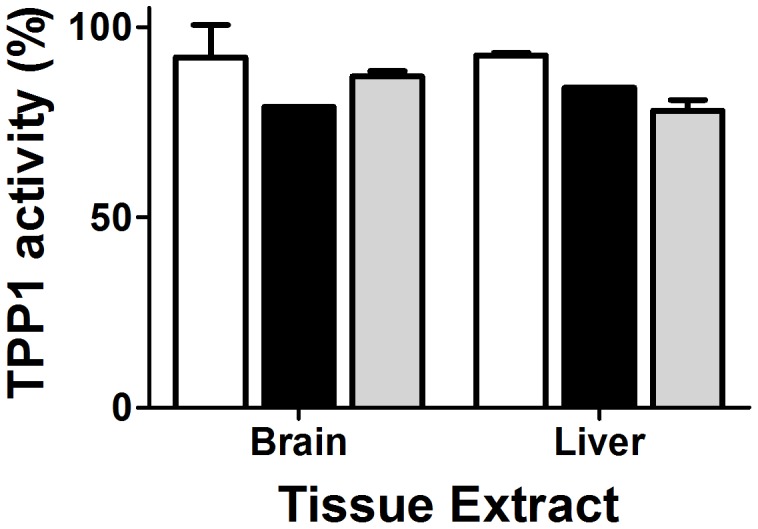
*In vitro* stability of TPP1 preparations in tissue extracts. Activated TPP1 was mixed with either brain or liver extracts (20 and 10 mg tissue wet weight/mL, respectively) from *Tpp1*(−/−) mice (final concentration 0.06 mg/mL TPP1, 90% tissue extracts in 150 mM sodium chloride, 100 mM sodium acetate pH 4.5, 0.1% Triton X-100), incubated at 37°C and frozen at the times indicated in Fig. 3 prior to measurement of TPP1 activity. Given that activity was relatively unchanged throughout the incubation, only the 24 h time point is shown. Unmodified TPP1, open bars; OX-TPP1, filled bars, DG-TPP1, gray bars. Error bars represent standard deviation from duplicate measurements.

It is possible that the glycans could help protect TPP1 from inactivation or degradation by plasma or lysosomal enzymes. To test this possibility, proTPP1 was incubated at neutral pH with plasma from TPP1-deficient mice and analyzed in terms of autoactivation and enzyme activity. Unmodified TPP1 was most stable with a half-life of ∼9 h, while the half-lives of OX-TPP1 and DG-TPP1 were decreased by ∼2.5 and 5-fold, respectively (Fig. 3). Analysis of the preparations by immunoblotting indicated that, under reducing conditions, all forms of TPP1 appeared stable (Fig. 4, Left Panel), migrating at the expected size of the TPP1 proenzyme monomer (see Fig. 1). Under non-reducing conditions, at zero time, TPP1 is present predominantly as a monomer with some dimer present (Fig. 4, Right Panel, asterisk). (Note that migration of the deglycosylated TPP1 monomer is distorted by the comigrating albumin band). Interestingly, incubation with plasma resulted in a time-dependent appearance of higher molecular weight immunoreactive material that was particularly pronounced for the modified preparations (Fig. 4, Right Panel, arrows). Intriguingly, TPP1 has one free cysteine that is completely buried in the protein core and that is located near the catalytic center [25]. It is possible that local unfolding makes this residue accessible and allows for thiol-disulfide interchange with plasma proteins, resulting in the irreversible inactivation of TPP1.

**Figure 6:**
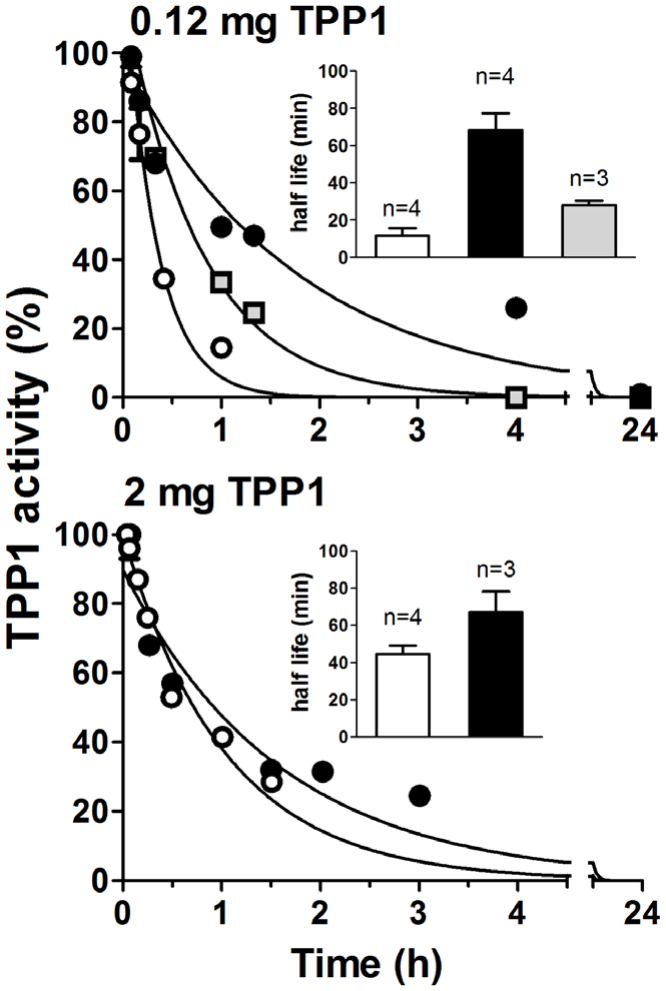
Plasma circulatory half-life of TPP1 preparations. *Tpp1*(−/−) mice were administered 0.12 mg (*Top*) or 2 mg (*Bottom*) of unmodified or modified TPP1 via tail vein injection and plasma samples collected via cheek puncture at indicated times. Unmodified TPP1, open circles; OX-TPP1, filled circles, DG-TPP1, gray squares. Plasma TPP1 activity was assayed after preactivation, normalized to the percentage of the activity at the initial time point and fit to a single phase exponential decay model constraining the plateau value to 0%. Curves show a single representative animal for each condition and insets represent mean and SEM for the indicated number of animals.

In addition, activated TPP1 was incubated with brain and liver extracts from TPP1-deficient mice under acidic conditions (pH 4.5). Under these conditions, there is considerable autolysis, suggesting this at least partially mimics the protease-rich environment of the lysosome (data not shown). All preparations retained >80% activity after 24 hours (Fig. 5). Taken together, these results suggests that glycans play a role in protecting the proTPP1 zymogen in plasma while they appear less critical for maintaining the activity of mature TPP1 in the lysosome.

**Figure 7:**
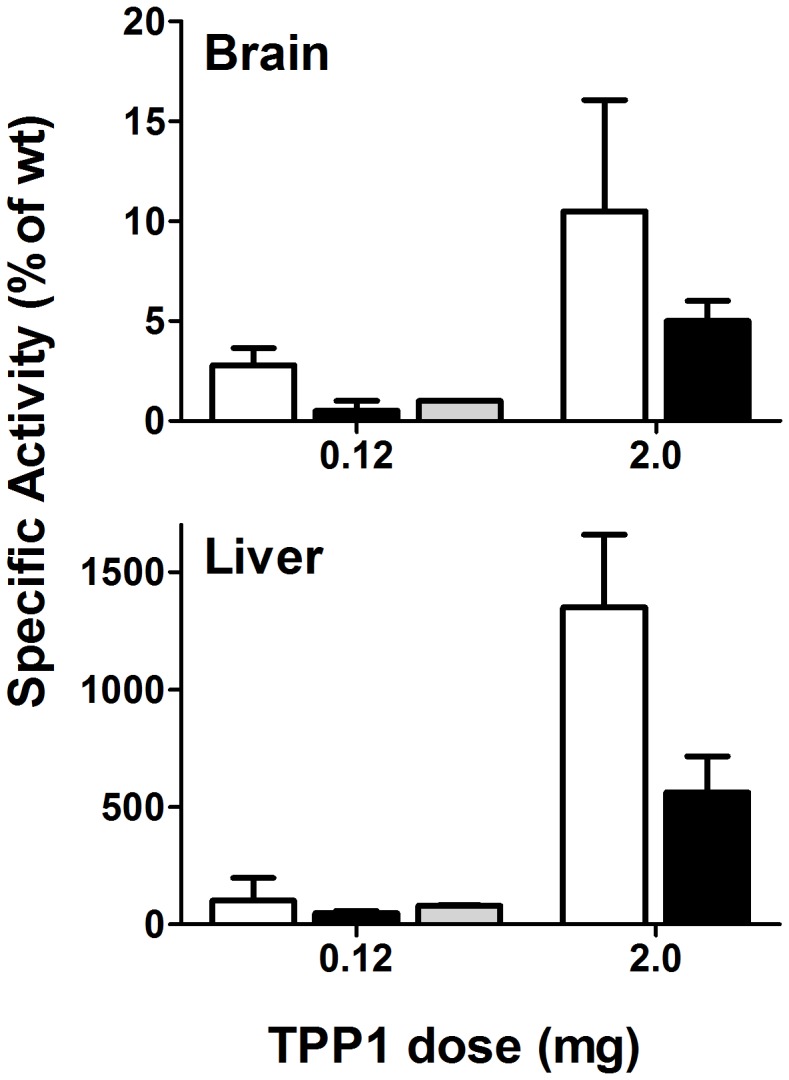
Biodistribution of TPP1 preparations. *Tpp1*(−/−) mice were administered indicated amounts of enzyme via tail vein injection and tissues collected after 24 hours. TPP1 specific activity (activity/mg protein) was normalized to that of wild-type controls. Unmodified TPP1, open bars; OX-TPP1, filled bars, DG-TPP1, gray bars. Sample size as in Table 1. Error bars represent standard deviation.

**Figure 8:**
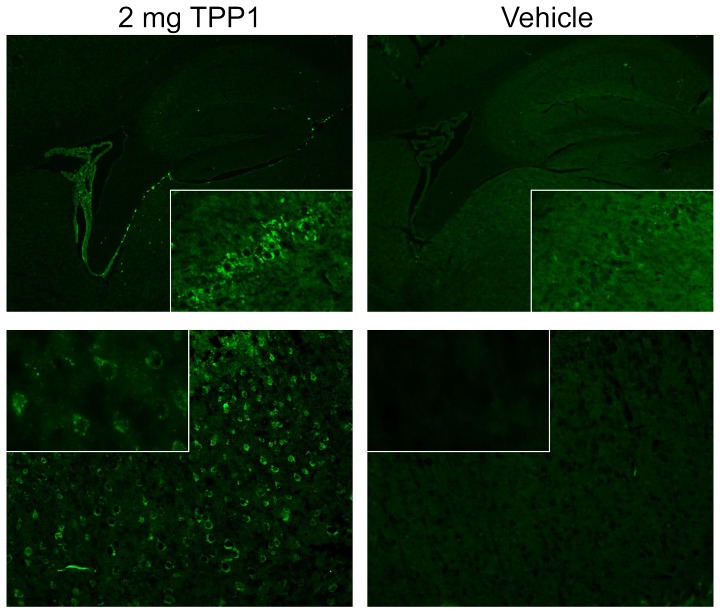
Immunostaining for TPP1. *Tpp1*(−/−) mice were administered unmodified enzyme or vehicle alone via tail vein injection and tissues collected after 24 hours. Tissue processing and immunostaining was conducted as described previously [33]. Top Panels show the choroid plexus and hippocampus (original magnification 4×) with inset showing hippocampal CA2 neurons (20×). Bottom Panels show cerebral cortex layer 2/3 (20× magnification) with inset showing select neurons (60×).

### Pharmacokinetics

To determine the effect of carbohydrate modification on clearance, we administered an IV bolus dose of each preparation to *Tpp1*(−/−) mice and measured TPP1 activity in blood samples taken at timed intervals. When administered at a dose of 0.12 mg (∼5 mg/kg), unmodified TPP1 was rapidly cleared from plasma, with a half-life of 12 min. OX-TPP1 had the longest half-life (∼70 min) while DG-TPP1 had an intermediate half-life (∼30 min) (Fig. 6). Note that the carbohydrate modification may result in increased association with plasma proteins and concomitant loss of activity (Figs. 3 and 4). Despite this, the prolonged plasma half-life for both modified preparations measured by TPP1 activity indicate that this is more than compensated for by the reduced clearance through Man6-P and other carbohydrate receptor mediated endocytic mechanisms. When administered at a higher dose (2 mg, ∼80 mg/kg), the plasma half-life of unmodified TPP1 increased to 45 min while that of OX-TPP1 was essentially unchanged (Fig. 6). This likely represents saturation of high-affinity, receptor-mediated endocytic pathways by the high concentrations of unmodified TPP1.

**Table 1:** Biodistribution of TPP1 preparations 24 hours following IV administration.

Genotype	*Tpp1*(+/+)	*Tpp1*(−/−)				
Preparation	-	Unmodified TPP1		OX-TPP1		DG-TPP1
Dose (mg)	-	0.12	2.0	0.12*	2.0*	0.12
Sample size	5	5	8	2	3	2
TPP1 specific activity, pmol/organ *(% of wild-type)*						
Brain	52.6±17.4 *(100%)*	1.4±0.4 *(3%)*	5.4±2.9 *(10%)*	0.3±0.4 *(1%)*	2.7±1.4 *(5%)*	0.7±0.1 *(1%)*
Liver	995±238 *(100%)*	1182±384 *(119%)*	13416±2811 *(1348%)*	361.8±56.3 *(36%)*	5357±802 *(538%)*	778.7±13 *(78%)*
Spleen	38.7±20.0 *(100%)*	43.9±45.4 *(113%)*	393.6±175.5 *(1017%)*	3.1±1.4 *(8%)*	108±22.6 *(279%)*	19.7±1.6 *(51%)*
Kidney	215±58.8 *(100%)*	17.4±14.5 *(8%)*	102±20.4 *(47%)*	7.0±3.2 *(3%)*	96.8±21.9 *(45%)*	10.1±1.9 *(5%)*
Heart	8.4±3.5 *(100%)*	3.1±2.8 *(37%)*	59.4±27.3 *(707%)*	2.7±0.6 *(32%)*	68.6±52.8 *(817%)*	1.2±0.3 *(14%)*
Lung	29.3±11.0 *(100%)*	5.7±6.6 *(19%)*	43.2±23 *(147%)*	3.1±3.1 *(11%)*	53±19.9 *(181%)*	1.5±1.4 *(5%)*
Sum of tissues *{% injected}*	1339	1253*{62%}*	14020*{42%}*	378*{23%}* *	5686*{21%}* *	812*{40%}*

* Only 82% of the oxidized proTPP1 could be autocatalytically converted to active enzyme. The dose represents total proenzyme based on concentration as determined by absorbance measurements, while % of injected activity recovered from all tissues analyzed is corrected for the amount of active enzyme (e.g., for OX-TPP1, pmol total injected*0.82).

Data are expressed as pmol TPP1 per organ and also compared to levels present in control *Tpp1*(+/+) mice. Note that for total recovery, the sum of the TPP1 detected in all tissues analyzed 24 hours after administration is compared to the administered dose without correcting for half-life. Data normalized to protein are presented in Table S1.

### Biodistribution

The primary goal of this study was to identify conditions that would result in improved targeting of IV administered TPP1 to the brain. To this end, TPP1 activity in brain and visceral tissues (liver, kidney, spleen, heart and lung) was analyzed 24 h after administration of enzyme. At any given dose, more of the unmodified TPP1 accumulated in brain compared to either modified form (Fig. 7, Table 1 and Table S1). The amount delivered to the brain was greater at the higher dose, with administration of 2 mg of unmodified TPP1 resulting in ∼10% of normal enzyme activity in the brain. Immunfluoresence analysis revealed specific punctate staining of TPP1 in neurons in the cerebral cortex and hippocampus although the most prominent staining was in the choroid plexus (Fig. 8). Thus, at least a portion of the administered TPP1 appears to reach neuronal lysosomes.

For all TPP1 preparations, among the tissues analyzed, ∼95% of the total activity recovered was associated with the liver. For the unmodified and DG-TPP1, this accounted for ∼40–60% of the administered dose (Table 1), while for OX-TPP1, only ∼20% was recovered. Given the ∼3 day half-life of recombinant human TPP1 targeted to the lysosome in different mouse tissues [5] and our finding that the active forms of all TPP1 preparations exhibited similar stability in tissue extracts under acidic conditions (Fig. 5), a substantial fraction of the OX-TPP1 may be taken up by other sites or cleared by other means. Alternatively, oxidation of carbohydrates may result in decreased stability following lysosomal delivery in ways not revealed by our *in vitro* analysis. Regardless, comparison of the the relative ratio of TPP1 activity in brain to liver reveals that compared to unmodified enzyme, OX-TPP1 and DG-TPP1 are unlikely to be preferentially delivered to the brain (Table 1).

## Discussion

The overall goal of this study was to determine whether IV administration of proTPP1 could result in significant targeting to the CNS. To this end, we examined both the effect of carbohydrate modification and the dose dependency of the enzyme on delivery to the brain.

Although carbohydrate modification of proTPP1 did increase its circulatory half-life, it did not improve targeting to the brain. It is not clear why β-glucuronidase and TPP1 differ in terms of the respective effects of carbohydrate modification on transit from the bloodstream to the brain but there are several possibilities. First, the carbohydrate modified forms of TPP1 were found to be less stable than the native form when incubated in plasma *in vitro*, which may help explain why the recovery of OX-TPP1 (∼20% of administered dose) was significantly less than that of native TPP1 (∼40–60%). Second, while the half-life of TPP1 could be increased significantly by chemical modification (∼6-fold, from 12 min to 70 minutes), the scale of this increase was considerably less than observed for β-glucuronidase (∼100-fold, from ∼12 min to ∼19 hours). It is possible that the greatly extended half-life is key to increased uptake to the brain. A third possibility is that β-glucuronidase is transported across the BBB via a pathway that is not effective for TPP1. This could, for example, reflect the fact that proteoglycans of the extracellular matrix, like heparan sulfate, are substrates for β-glucuronidase [27] and may be bound by the protein in circulation. In doing so, this would increase the local concentration on the surface of the endothelial cells, possibly mediating uptake via endocytic or pinocytic routes.

It is likely that each lysosomal protein will exhibit differences in targeting to the brain and will respond differently to carbohydrate modification. It has recently been reported that modification of sulfamidase increases its circulatory half-life and targeting to the brain, although this appeared ineffective in terms of delivery to neurons, with protein only being detected in blood-associated compartments [28]. In contrast, TPP1 was similar to β-glucuronidase in terms of reaching neurons following intravenous administration.

Both unmodified and OX-TPP1 displayed a dose-dependent increase in targeting to the brain, with the unmodified form being superior. Note that while the absolute amount of TPP1 in brain increased with increasing doses (3 to 10% wild-type activity for the 0.12 and 2 mg doses of unmodified TPP1, respectively), the percentage of administered enzyme actually decreased (16-fold increase in dose compared to a 3-fold increase in brain). This suggests that adjustments in the dosing parameters (e.g., prolonged infusion of low concentrations of enzyme) may improve efficacy of delivery. We are currently exploring this as well as the therapeutic efficacy of IV administration in the LINCL mouse model.

## Materials and Methods

### Ethics Statement

All experiments and procedures involving live animals were conducted in compliance with protocols approved by the Robert Wood Johnson Medical School Institutional Animal Care and Use Committee (“Preclinical evaluation of therapy in an animal model for LINCL”, protocol I09-0274-4). All efforts were made to minimize suffering.

### Animals and tissue collection

The *Tpp1*(−/−) mouse model for LINCL was in a C57/BL6 strain background and genotyped as described [29]. TPP1 was administered by tail vein injection. Blood was sampled by cheek puncture and collected into heparin-coated capillary tubes and centrifuged at 1,000× g for 15 min to yield plasma. Animals were killed by transcardial perfusion using saline after anesthetization with 98 mg/ml sodium pentobarbital containing 12.5 mg/ml phenytoin (a 1∶4 dilution of Euthasol; Delmarva Laboratories, Midlothian, VA). In some cases, plasma was obtained by heart puncture and collection into heparin-containing tubes. Mice were 70 to 90 days old at the time of analysis. Tissues from saline-perfused mice were snap frozen on dry ice, pulverized using a Bessman homogenizer, and stored at −80°C.

### Production of TPP1

Recombinant human TPP1 proenzyme was produced in Chinese hamster ovary (CHO) cells and purified as described previously [30]. The purified enzyme was concentrated to 10 mg/ml in an artificial CSF buffer (aCSF; 148 mM NaCl, 3 mM KCl, 1.4 mM CaCl_2_, 0.9 mM MgCl_2_, 0.8 mM Na_2_HPO_4_, 0.196 mM NaH_2_PO_4_, pH 7.2) and stored at −80°C. Protein concentration of the proenzyme was estimated by measuring absorbance at 280 nm using an extinction coefficient of 82,195 M^−1^ cm^−1^ (1.38 ml mg^−1^ cm^−1^).

### Chemical modification of TPP1

Chemical modification of exposed carbohydrates of TPP1 (resulting in the form designated OX-TPP1) was conducted by oxidative degradation with sodium metaperiodate followed by reduction with sodium borohydride using a modification of published methods [22]. In brief, TPP1 in aCSF was buffer exchanged to 20 mM sodium phosphate, 20 µM CaCl_2_, 150 mM NaCl, pH 6.0 using a PD-10 column (GE Healthcare). All reactions were performed at 0–4°C in the dark. Sodium periodate was added to a final concentration of 20 mM and TPP1 was incubated for 6.5 h. The reaction was terminated after by addition of ethylene glycol to a final concentration of 200 mM. After 15 min incubation, the TPP1 was buffer-exchanged into 20 mM sodium phosphate, 20 µM CaCl_2_, 150 mM NaCl, pH 8.0 by PD-10 chromatography. Sodium borohydride was added to a final concentration of 100 mM and TPP1 was incubated for ∼12 h. TPP1 was buffer exchanged to aCSF by PD-10 chromatography and purified by gel filtration chromatography on a Superdex 75 column. Fractions containing TPP1 were pooled, concentrated to 8 to 10 mg/ml and aliquots stored at −80°C. Detection of carbohydrates containing mannose 6-phosphate was conducted using an iodinated soluble form of the bovine cation-independent mannose 6-phosphate receptor [31].

### Deglycosylation of TPP1

Production and purification of deglycosylated TPP1 (designated DG-TPP1) was as previously described [25]. In brief, high mannose TPP1 produced from CHO cells grown in the presence of kifunensine was purified by butyl-Sepharose 4 fast-flow and Mono Q12 chromatography, digested with Endoglycosidase Hf (Endo H; 26,500 units per mg of pro-TPP1, New England Biolabs) at 25°C for 15 h followed by 1 h at 37°C and further purified by Mono Q12 and Superdex 75 chromatography. Protein was concentrated to 1 mg/mL in aCSF and aliquots stored at −80°C.

### TPP1 assays

Purified proenzyme and plasma samples were incubated at pH 3.5 to convert the inactive zymogen to active enzyme prior to measuring TPP1 activity at pH 4.5 using the Ala-Ala-Phe-AMC substrate as described previously [32]. A reference standard of purified, preactivated TPP1 was analyzed in parallel with experimental samples, allowing for the calculation of pmol equivalents of active TPP1. Frozen tissue powders were homogenized in 150 mM sodium chloride, 100 mM sodium acetate pH 4.5, 0.1% Triton X-100 and a cleared supernatant prepared by centrifugation (13,000× g for 30 min at 4°C). No preactivation step was used when measuring TPP1 activity as control experiments on liver extracts from animals injected with all forms of TPP1 indicated that preactivation did not increase the activity. In addition, control experiments where liver powders from enzyme-treated *Tpp1(−/−)* animals were subjected to ten freeze-thaw cycles revealed no loss of activity for either modified or unmodified TPP1 preparations. The protein content of tissue extracts were measured using Advanced Protein Assay (Cytoskeleton Inc.) with bovine serum albumin standards. Denaturing polyacrylamide gel electrophoresis (SDS-PAGE) was conducted on 10% NuPAGE MOPS gels (Invitrogen). Immunoblotting and immunofluorescence was as described previously [33].

## Supporting Information

Table S1 **Biodistribution of TPP1 preparations 24 hours following IV administration.** Data are expressed as pmol TPP1 per mg protein and also compared to levels present in control *Tpp1*(+/+) mice.
(DOCX)
