# Supplementary material for: Systemic Administration of Tripeptidyl Peptidase I in a Mouse Model of Late Infantile Neuronal Ceroid Lipofuscinosis: Effect of Glycan Modification
Source: PLoS One. 2012 Jul 6;7(7):e40509. doi: 10.1371/journal.pone.0040509 (PMC3391252; doi:10.1371/journal.pone.0040509)
Supplement: Table S1 — Biodistribution of TPP1 preparations 24 hours following IV administration. Data are expressed as pmol TPP1 per mg protein and also compared to levels present in control Tpp1(+/+) mice. (DOCX) [file pone.0040509.s001.docx]

**Supplemental Table 1. Biodistribution of TPP1 preparations 24 hours following IV administration.** Data are expressed as pmol TPP1 per mg protein and also compared to levels present in control *Tpp1*(+/+) mice.

| Genotype | *Tpp1*(+/+) | *Tpp1*(-/-) | | | | |
| --- | --- | --- | --- | --- | --- | --- |
| Preparation | - | Unmodified TPP1 | | OX-TPP1 | | DG-TPP1 |
| Dose (mg) | - | 0.12 | 2.0 | 0.12 | 2.0 | 0.12 |
| Sample size | 5 | 5 | 8 | 2 | 3 | 2 |
|  | TPP1 specific activity (pmol/mg protein)  (% of wild-type) | | | | | |
| Brain | 2.18±0.45  (100%) | 0.06±0.02  (3%) | 0.23±0.13  (11%) | 0.01±0.02  (1%) | 0.11±0.04  (5%) | 0.03±0.001  (1%) |
| Liver | 5.80±0.94  (100%) | 5.84±5.62  (101%) | 78.3±18.0  (1349%) | 2.78±0.54  (48%) | 32.63±8.86  (562%) | 4.64±0.06  (80%) |
| Spleen | 5.76±1.48  (100%) | 6.16±3.27  (107%) | 57.6±11.7  (1001%) | 0.88±0.26  (15%) | 14.52±4.30  (252%) | 2.98±0.19  (52%) |
| Kidney | 7.46±1.54  (100%) | 0.51±0.27  (7%) | 3.21±0.78  (43%) | 0.23±0.06  (3%) | 3.20±0.66  (43%) | 0.33±0.04  (4%) |
| Heart | 1.12±0.19  (100%) | 0.45±0.46  (40%) | 8.33±3.90  (746%) | 0.42±0.05  (38%) | 7.30±1.57  (654%) | 0.24±0.03  (21%) |
| Lung | 4.78±0.52  (100%) | 0.43±0.37  (9%) | 6.44±2.75  (135%) | 0.31±0.12  (6%) | 5.46±1.11  (114%) | 0.23±0.06  (5%) |
